# Deterrence of birds with an artificial predator, the RobotFalcon

**DOI:** 10.1098/rsif.2022.0497

**Published:** 2022-10-26

**Authors:** Rolf F. Storms, Claudio Carere, Robert Musters, Hans van Gasteren, Simon Verhulst, Charlotte K. Hemelrijk

**Affiliations:** ^1^ Groningen Institute for Evolutionary Life Sciences, University of Groningen, Groningen, The Netherlands; ^2^ Department of Ecological and Biological Sciences, University of Tuscia, Viterbo, Italy; ^3^ Roflight, Lemselobrink 32, 7544 GD Enschede, The Netherlands; ^4^ Royal Netherlands Air Force, Breda, The Netherlands

**Keywords:** RobotFalcon, deterrence, birds, habituation, predation

## Abstract

Collisions between birds and airplanes can damage aircrafts, resulting in delays and cancellation of flights, costing the international civil aviation industry more than 1.4 billion US dollars annually. Driving away birds is therefore crucial, but the effectiveness of current deterrence methods is limited. Live avian predators can be an effective deterrent, because potential prey will not habituate to them, but live predators cannot be controlled entirely. Thus, there is an urgent need for new deterrence methods. We developed the RobotFalcon, a device modelled after the peregrine falcon, and tested its effectiveness to deter flocks of corvids, gulls, starlings and lapwings. We compared its effectiveness with that of a drone, and of conventional methods routinely applied at a military airbase. The RobotFalcon scared away bird flocks from fields immediately, and these fields subsequently remained free of bird flocks for hours. The RobotFalcon outperformed the drone and the best conventional method at the airbase (distress calls). Importantly, there was no evidence that bird flocks habituated to the RobotFalcon over the course of the fieldwork. We conclude that the RobotFalcon is a practical and ethical solution to drive away bird flocks with all advantages of live predators but without their limitations.

## 1. Introduction

Flocks of birds are known to conflict with human activities in a multitude of areas and contexts. In agriculture, gregarious birds eating crops cause economic damage [1]. In urban environments, bird flocks may damage buildings with their nests, be a potential spread of disease and cause discomfort by harassing people [2–4]. A major area where problems arise with birds is aviation: Birds colliding with aircrafts (i.e. bird strikes), cost the civil aviation industry more than 1.4 billion US dollars annually [5–7], and in the last century bird strikes have led to over 450 deaths in military aviation alone [8–10]. Thus, birds cause non-negligible economic loss and safety hazards and the risk is heightened due to the flocking behaviour of many species [11].

To reduce these societal costs, it is necessary to deter birds from specific locations. Many ways to do so have been explored. Habitats have been made unattractive to some species of birds as a preventive measure. Despite this, some aspects of these habitats may remain attractive and certain bird species may use the them as a stopover [12,13]. Corrective measures are applied to further reduce the bird numbers. The main methods rely on acoustic (distress calls and pyrotechnics) or visual deterrents (dogs, falcon silhouettes and scarecrows) and trapping and relocating birds [15,16]. Some methods are harmful, e.g. blinding birds with a laser [14], or killing them (live shooting and falconry [15]). No method can clear areas from birds indefinitely and the time until birds return varies per method. Most methods suffer from some degree of habituation: after repeated exposure, birds respond less [17]. Given the variable and temporary effectiveness of available methods there is an urgent need for new and more effective methods.

Habituation is expected to be reduced when deterrence methods resemble natural threats, such as falconry [15,18,19]. However, breeding and training falcons is very costly, and the effectiveness of falconry is limited because falcons cannot be flown often and guiding their attacks is problematic [15,20]. Instead of live falcons, models that mimic predators visually and behaviourally may be a promising way to deter birds (e.g. [21]), retaining the advantages of a live predator, but with fewer practical limitations. We therefore developed an artificial raptor, the RobotFalcon, inspired specifically by a peregrine falcon (*Falco peregrinus*). This species hunts a wide spectrum of bird species over a large part of the globe and its hunting behaviour is well studied (e.g. [22–24]). The RobotFalcon closely resembles the peregrine falcon in its shape, the coloration of its wings, beak and head, its overall size and its relative dimension of wing and tail (figure 2*a*). It has the advantage that it can be precisely steered to target a flock and can be flown more frequently than live falcons. The RobotFalcon can be steered from its own perspective via a camera on its back (figure 2*c*, first-person view).

In this field study, we tested the effectiveness of the RobotFalcon to drive away bird flocks by measuring the proportion of flocks it drove away, how fast fields were cleared from flocks, how long it took for them to return, and whether habituation occurred. To this end, the RobotFalcon was flown on several bird species in an agrarian environment (Workum, The Netherlands). The behaviour of the bird flocks was studied upon exposure to the RobotFalcon, to a normal drone and in control trials without any disturbance. We further compared the effectiveness of the RobotFalcon with the conventional methods in current use at a military airport such as distress calls and pyrotechnics.

## 2. Material and methods

### 2.1. Study area

**Figure 1.**
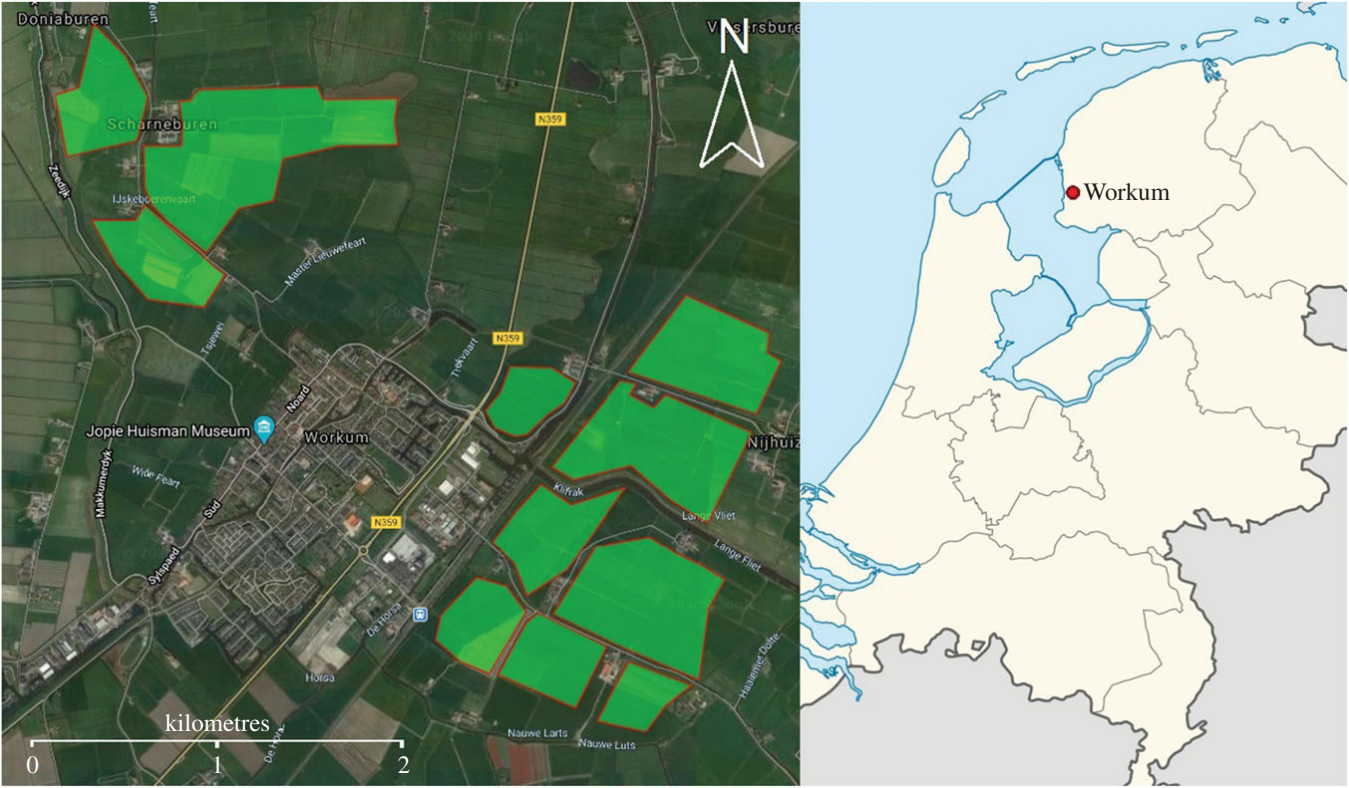
The research fields used for experiments in Workum, highlighted in green.

The fieldwork was carried out in the agricultural area surrounding Workum, The Netherlands (52°59' N–5°27' E, figure 1). There was no significant variation in elevation within the area. Flights with the RobotFalcon and drone were carried out at least 100 m from buildings and trees, allowing us to keep track of them throughout their flights as well as minimizing any impact of landscape characteristics on the behaviour of the birds. The hunting actions of the RobotFalcon were focused on corvids (*Corvus monedula*, *Corvus frugilegus* and *Corvus corone*), gulls (*Chroicocephalus ridibundus* and *Larus canus*), northern lapwings (*Vanellus vanellus*) and starlings (*Sturnus vulgaris*). These species are common in the study area and frequently conflict with human activities and flight safety on aerodromes [2,20,25].

### 2.2. RobotFalcon and drone

**Figure 2.**
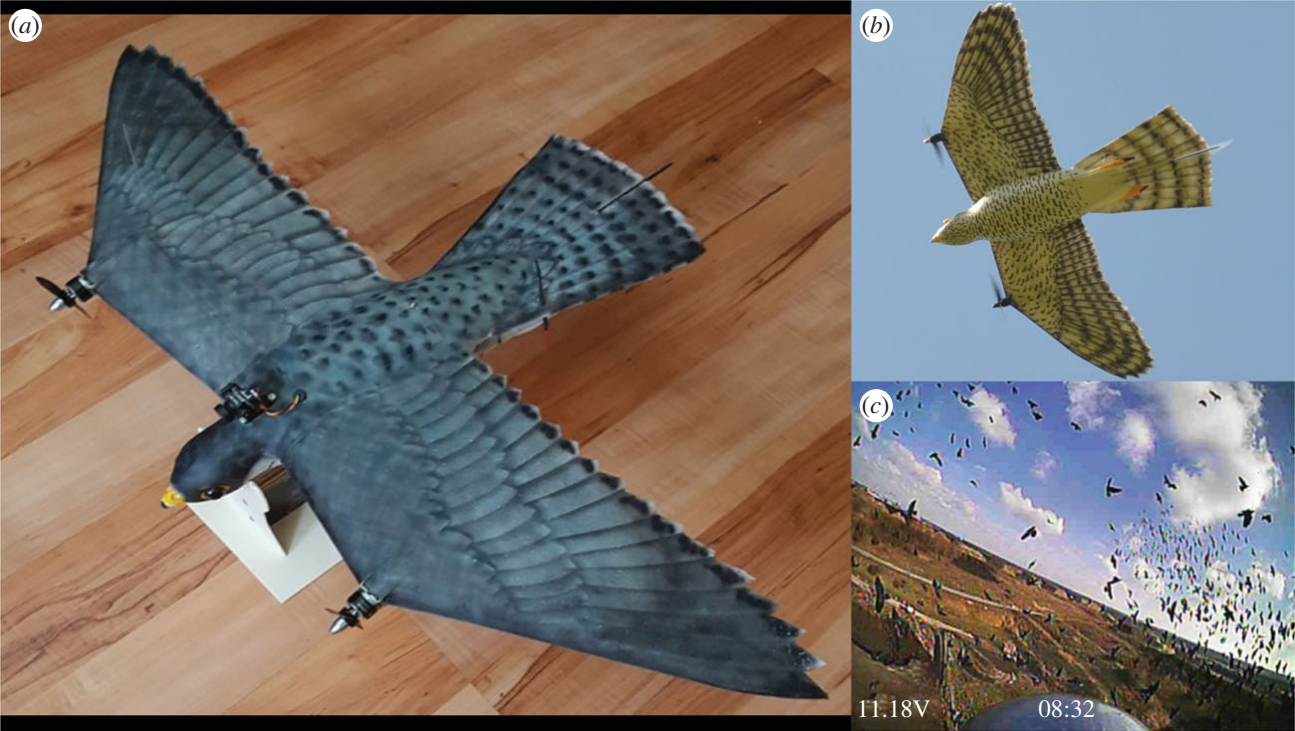
The RobotFalcon (*a*), a view from the RobotFalcon's underside during flight (*b*) and an example of its view during flight (*c*).

The RobotFalcon was developed by one of the authors (R.M.). Its coloration, shape, overall size and the relative dimension of wing and tail were customized to closely resemble a peregrine falcon, *Falco peregrinus* (figure 2). Its body is made of fibreglass, and its wings and tail are made of expanded polypropylene, reinforced with carbon fibre. The parts were coloured by air-brush. The RobotFalcon weighs 0.245 kg and has a wingspan of 70 cm. It has two propellors, one on each wing, with additional control surfaces on the tail for steering and has a cruise speed of 15 m s^−1^. The wings do not flap, which allows for greater controllability/steerability during the flight. A camera (Runcam micro swift2, 30 fps) on the head enables first-person view while steering. Two certified operators (R.M. and R.W.) steered the RobotFalcon alternatingly. Controlling for pilot identity in no case changed the results of the statistical analyses. For simplicity, we therefore excluded this factor from the analysis presented in the paper.

A DJI Mavic Pro drone lacking any raptor features was used for comparison. The drone was black, weighed 0.734 kg, had a diagonal length of 335 mm and a maximum speed of 18 m s^−1^ (electronic supplementary material, figure S1).

### 2.3. Field procedure

Field work was done on 34 days between 25 February 2019 and 22 November 2019, excluding the breeding season (April to July) carrying out on average six field days a month. The experiments were conducted by a team of three people: a pilot, an operator of a ground camera (Sony FDR-AX53 4K Camcorder, 50 fps) and a coordinator with audio recorder and GPS receiver. The speed and direction of the wind were measured immediately prior to the flights, using an anemometer (Kaindl Windmaster 2) and a compass (Compass Galaxy). We avoided rain and strong wind (greater than 6 on the Beaufort scale). We recorded which birds were present (species and number), their behaviour (foraging, resting or restless) and location (using a Bushnell Tour V4 range finder and the ground camera).

When flocks of the aforementioned species were spotted on the ground, a deterrence experiment started. Twenty-five per cent of these experiments were randomly assigned to start with a control trial (see Results for details). During these trials, birds were monitored without performing any deterrence action for ten minutes.

If flocks of birds remained after a control trial or if no control trial was assigned, a deterrence action was performed with either the RobotFalcon or the drone (chosen randomly). A deterrence action included the whole sequence of the RobotFalcon or the drone approaching the flock until taking off and subsequently hunting the flock until it was out of sight. At the start, the pilot flew the RobotFalcon or the drone such that it approached the birds in a straight line at a constant altitude, until the birds initiated flight. The altitude of this approach was randomly determined to be either high (greater than 50 m) or low (less than 50 m), both with a probability 0.5. We chose 50 m as the threshold. If the threshold was higher, the pilot could no longer distinguish birds on the ground. When approaching birds from a low altitude, we aimed to have the model predator fly as low as possible (e.g. 5–20 m), and actual altitude was measured through the GPS in the RobotFalcon. In practice, due to limited altitude feedback to the pilot, there was substantial variation in altitude within flights intended to be high and low and in the statistical analysis we therefore used the actual altitude rather than the categories high and low (see below).

The flight initiation of the flock was defined as the moment when at least one bird started taking off (i.e. from the moment it started flapping its wings) and was followed by the rest of the flock. Once the flock was airborne, the RobotFalcon or the drone chased it (pursuit), while occasionally trying to intercept individuals by diving in the flock (attacks). This mimicked the hunting behaviour of real peregrine falcons, following videorecordings and behavioural analyses in previous work [22,24].

Throughout the deterrence action, the behaviour of the birds was recorded with the ground camera and audio recordings. A hunting sequence was considered successful when the birds flew away over a distance beyond 1 km, which in most cases implied that they were out of view (using 8 × 40 binocular). After this, we monitored the experimental area at intervals of 30 min for up to 120 min in order to record return times of birds of the same species.

### 2.4. Data collection and analysis

Footage from the ground camera was synchronized with the GPS data of the RobotFalcon using Adobe Premiere Pro, and analysed manually on a frame by frame basis, recording the escape of the flocks.

Deterrence success was quantified in two ways: firstly, by the proportion of deterrence actions that cleared fields from bird flocks and, secondly, by the duration the fields remained clear of bird flocks after deterrence. Flocks were counted to have returned when more than five individuals of that species were observed on the site. Further, we measured the frequency of collective escape responses of the flocks when airborne (e.g. blackening, splitting, flash expansions; see [24]).

The latitude, longitude and altitude of the position of the RobotFalcon at the beginning of the flight response were used to estimate the distance between the flock of birds (using the location of the flockmember closest to the RobotFalcon, measured with a rangefinder) and the RobotFalcon: the flight initiation distance (FID). Since the drone needed to approach a flock several times before it took flight, while in a number of cases birds did not fly up at all, we did not measure the FID of a flock to the drone. Instead, we compared between the drone and RobotFalcon by counting the number of times birds landed during a flight as a measure of reluctance to stay airborne.

Statistical analyses were carried out in R [26]. The effectiveness of the RobotFalcon was compared to that of the drone and of deterrence methods applied at the military airbase Leeuwarden. Deterrence data from airbase Leeuwarden were collected from 2001 to 2016, involving methods such as bioacoustics and pyrotechnics. The proportion of deterrence actions that resulted in clearing the field of birds was compared between the RobotFalcon and drone using a two-way ANOVA. A survival analysis was performed on the time it took the flock to return.

Variation of FID over time was analysed with generalized linear mixed models taking into account the species, the approach altitude of the RobotFalcon and weather conditions as fixed effects, and flight identity as random effect to account for the non-independence of data on multiple species deterred during a given flight.

## 3. Results

**Figure 3.**
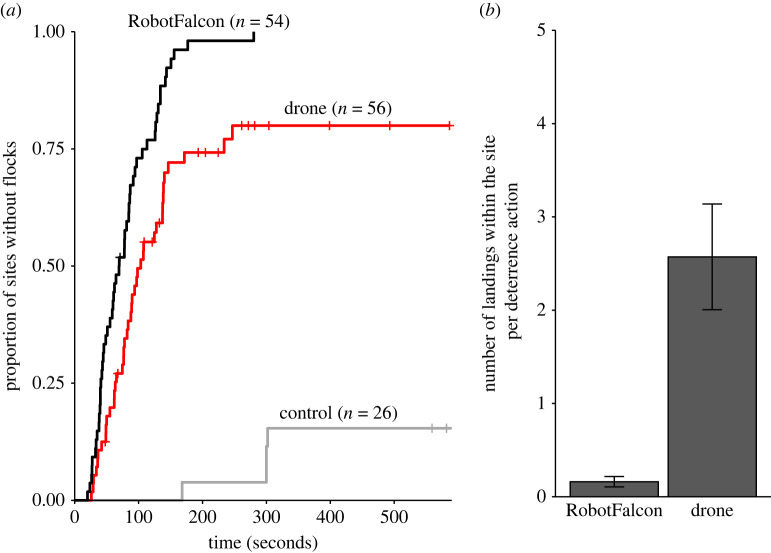
Flock responses to experimental and control flights (= no disturbance). (*a*) Proportion of fields cleared from flocks of birds over time after being approached by the RobotFalcon, drone or neither (control session). The three methods differed significantly ($\chi_{2,N\;=\;136}^{2}\;=\;70.7$ , *p* < 0.001). (*b*) The average number of times flock members landed again after flying up for the RobotFalcon and drone (±s.e.m.). Flocks landed again at the field significantly more often after flying up for the drone than the RobotFalcon (*t*_56_ = 4.23, *p* < 0.01).

All flocks were successfully deterred by the RobotFalcon within five minutes after it started its flight, with 50% of deterrence flights resulting in fields being free of birds within 70 s (54 flocks, figure 3*a*), while in the control sessions, without deterrence, 15% of locations were free of birds after 5 min (26 flocks, figure 3*a*).

With the drone, it took longer to clear fields from flocks, and fewer fields were cleared: half of the fields were cleared after 100 s and 80% after 5 min (56 flocks, figure 3*a*). The RobotFalcon was more effective in keeping flocks airborne than the drone: brief occasional landings of flocks after taking flight were less frequent when deterring with the RobotFalcon (*M* = 0.2 landings per hunt, s.e. = 0.06) than with the drone (*M* = 2.6 landings per hunt, s.e. = 0.6; figure 3*b*).

**Figure 4.**
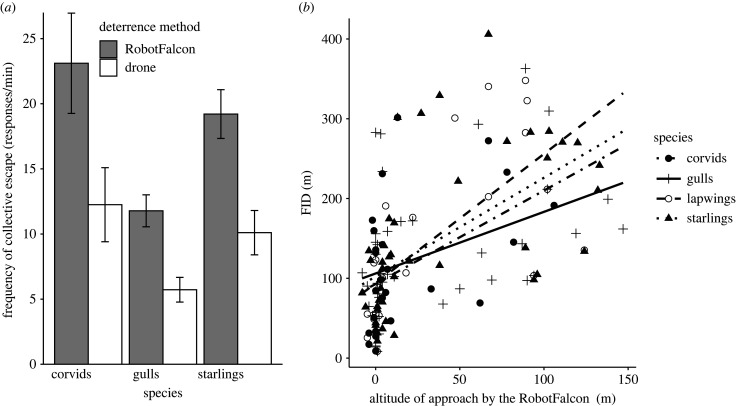
Collective escape of flocks of corvids, gulls and starlings when chased artificially. (*a*) The frequency of collective escape from the RobotFalcon and the drone (±s.e.m.). (*b*) The higher the approach altitude of the RobotFalcon, the further the distance at which flocks initiated flight (FID).

As regards species differences, the RobotFalcon chased away flocks of corvids and gulls significantly faster than the drone, while starlings were chased away by both methods equally fast (electronic supplementary material, figure S2). Flocks of all species displayed more often patterns of collective escape in response to the RobotFalcon than to the drone (figure 4).

When the RobotFalcon approached from a higher altitude, flocks of all species fled sooner (figure 4*b*).

**Figure 5.**
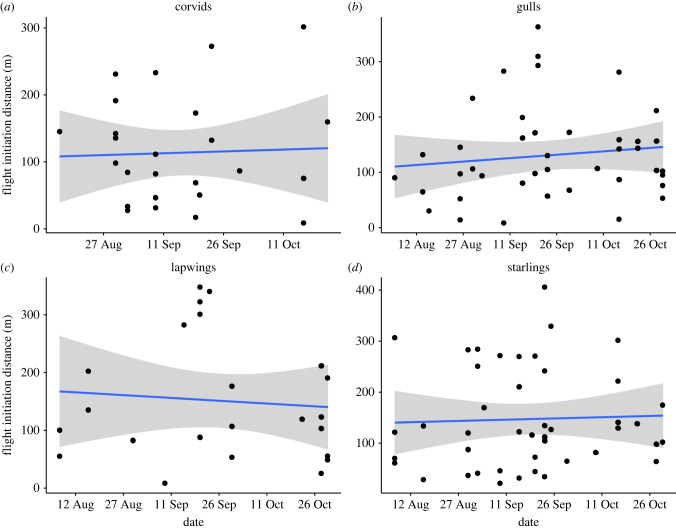
The absence of change in the distance at which birds flocks initiated flight (FID) in response to the RobotFalcon over the period of three months of fieldwork in Workum, The Netherlands. Habituation would have resulted in a decrease of FID over time.

Over the course of our fieldwork the success of the RobotFalcon at clearing fields remained high and the FID of the flocks did not change for any of the species (figure 5).

**Figure 6.**
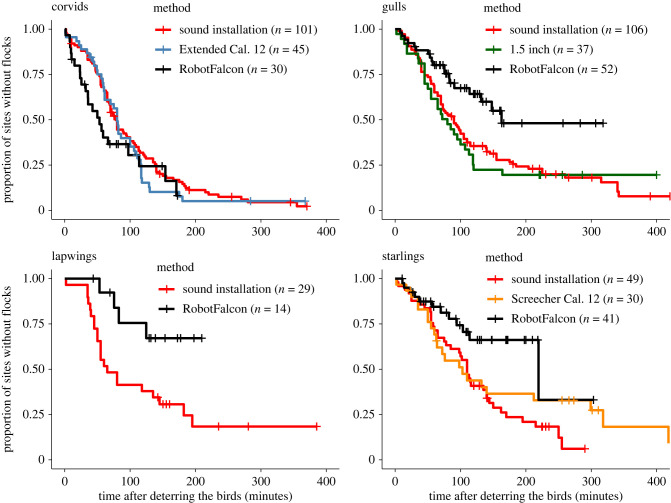
Proportion of fields without birds after deterrence with the RobotFalcon or other methods. Proportion of fields that was without birds over time after flocks of corvids, gulls, lapwings and starlings were chased away. For the airbase Leeuwarden, we show only the results for the method with the best results for each species. The sound installation method involves playing back distress calls of the species under concern. The Extended Cal. 12, 1.5 Inch and Screecher Cal. 12 are all variants of pyrotechnics. Gulls, lapwings and starlings stayed away significantly longer when chased away with the RobotFalcon than with distress calls ($\chi_{2,N\;=\;195}^{2}\;=\;10.4$, *p* = 0.006; $\chi_{1,N\;=\;43}^{2}\;=\;5.9$, *p* = 0.02; $\chi_{2,N\;=\;120}^{2}\;=\;8.3$, *p* = 0.02). Corvids stayed away equally long when chased by either method ($\chi_{2,N\;=\;176}^{2}=\;2.6$, *p* = 0.3).

Compared to the method that cleared fields of flocks for the longest period at airbase Leeuwarden (distress calls and pyrotechnics), the RobotFalcon caused flocks of gulls, lapwings and starlings to stay away longer (figure 6). More specifically, in response to the RobotFalcon, flocks of starlings and lapwings stayed away for a median time of 4 h, compared to 1.83 and 1.1 h, respectively, when deterred by distress calls. Flocks of gulls stayed away for a median time of 3 h after flights with the RobotFalcon versus 1.5 h when scared by distress calls. Corvids stayed away equally long when deterred by the RobotFalcon and distress calls (about an hour for both methods, figure 6).

## 4. Discussion

There is a need for novel methods to deter birds, and we show that the RobotFalcon can make a major contribution to filling that niche. It cleared fields from corvids, gulls, starlings and lapwings successfully and fast, with deterred flocks staying away for hours. The RobotFalcon was more effective than a drone: its success was higher, and it deterred flocks faster. The effectiveness of the RobotFalcon was similar across flocks of different species, while that of the drone was lower for flocks of gulls and corvids than for starlings. Starlings might be more inclined to flee because of their smaller size. Red-winged blackbirds, which are similarly sized to starlings, have also been found to fly away from low approaching drones [27]. The effectiveness of the RobotFalcon was higher when it approached flocks from a higher altitude, as shown by the longer FID. This may be because it represents a greater potential threat if it approaches from above, or because it is detected earlier by the flock. We compared the RobotFalcon against the most effective methods used at the airbase Leeuwarden: distress calls and pyrotechnics. The RobotFalcon kept away flocks of gulls, lapwings and starlings (but not corvids) for longer than the best methods at the airbase Leeuwarden. Fields were kept free from corvids equally long when the flocks were deterred by the RobotFalcon or the best airbase methods. This may due to the stronger dependance of corvids on local resources than gulls, lapwings and starlings. A limitation of our approach is that we compared different methods at different sites (RobotFalcon in Workum versus best airbase methods in Leeuwarden). This comparison is conservative however, because even though the habitat management by airbase Leeuwarden made their area less attractive to birds, flocks still returned to these areas sooner than to our fields in Workum.

Our study shows for the first time that a RobotFalcon, modelled after a peregrine falcon, effectively deters flocks of several species of birds in their natural environment. Previous studies showing the escape from models that mimic a real predator have all been conducted in captivity (in fish [28]; in insects [29]; in birds [21]). Besides, experiments on escape from a predator model in birds concerned only single birds, not the escape of a flock [21].

Effectiveness of most of the current methods to drive away birds is reduced by habituation, with birds fleeing less over time. Birds habituate in particular to methods that do not represent a natural threat (such as synthetic sounds, gas cannons and reflectors), especially when such methods are the only ones used in the field [15,30–33]. Royal Netherlands Air Force resolves this by alternating between different methods (species specific distress calls of birds and pyrotechnics). This alternation prevents habituation, but birds return sooner still than when chased away by the RobotFalcon.

In our three months of fieldwork, there was no evidence of habituation of birds to the RobotFalcon. We speculate that the RobotFalcon continued to be effective because of its resemblance in behaviour and appearance to a real falcon.

This may also explain why the RobotFalcon performed better than the drone. Since we were unable to follow birds individually, however, the lack of habituation we recorded could be either caused by us deterring naive birds each day due to the turnover of the bird population, or it may reflect an actual lack of habituation of individual birds. We cannot distinguish between these options, but it is likely that both processes have contributed to the observed pattern. We emphasize that for practical purposes the salient finding is that there was no decrease in success by the RobotFalcon in clearing fields over the three months of our fieldwork. For measuring actual levels of habituation to the RobotFalcon, specific experiments in more controlled conditions in truly resident bird populations such as domestic pigeons should be carried out.

A question remains as to what specifically made the flocks respond more to the RobotFalcon than to the drone: was this due to the falcon-like silhouette or due to the falcon-like coloration? Further studies are needed to disentangle this, for instance by revising either coloration (painting the model black) or morphology (while retaining the coloration). Notably, we aimed to mimic the hunting strategy of a real peregrine falcon when deterring birds with both the RobotFalcon and drone. To what degree did this impact the response of the birds and was the drone as successful in replicating this behaviour as the RobotFalcon? There was a size difference between the DJI drone and the RobotFalcon, with the wingspan of the RobotFalcon exceeding the diagonal length of the drone (excluding rotors), which may also have contributed to the difference in response.

Some studies have combined drones with natural stimuli such as distress calls and taxidermied crows, indirectly indicating the presence of a predator, to drive away birds [34–36]. It would be interesting to combine distress calls and bird taxidermy with a RobotFalcon to test whether this makes for an even more effective scaring device.

While the RobotFalcon has proven to be a highly effective tool to deter birds, it is important to also recognize its limitations, which are that steering the RobotFalcon requires trained pilots, flights are limited by battery life (15 min per battery) and cannot be conducted under rain or strong wind conditions. Further, to deter large birds successfully such as geese or herons, the RobotFalcon might be not effective enough and a robot that mimicks a natural (larger) predator of large-sized birds, e.g. an eagle, should be developed and tested. Deterrence with the RobotFalcon can, however, replace falconry, because it has the same advantages but not the limitations of live birds of prey.

In conclusion, the RobotFalcon provides a method to effectively deter flocks of a wide range of bird species, with no signs of habituation, making it a valuable addition to the tool-box currently available.

## Data Availability

The data that support the findings of this study are uploaded to the 4TU Research Data repository and available online: https://doi.org/10.4121/21256368.v1 [37]. The data are provided in electronic supplementary material [38].
